# Cyber victimization and well-being in adolescents: The sequential mediation role of forgiveness and coping with cyberbullying

**DOI:** 10.3389/fpsyg.2022.819049

**Published:** 2022-11-18

**Authors:** Yüksel Eroglu, Adem Peker, Serkan Cengiz

**Affiliations:** ^1^Psychological Counseling and Guidance, Afyon Kocatepe University, Afyonkarahisar, Turkey; ^2^Psychological Counseling and Guidance, Atatürk University, Erzurum, Turkey

**Keywords:** cyber victimization, well-being, forgiveness, mediation, coping with cyberbullying

## Abstract

Cyber victimization is an important problem among adolescents and it can have negative effects on well-being. However, efforts to increase the well-being of cyber victims have been increasing in recent years. It is important to uncover the underlying mechanisms that may affect the well-being of cyber victims. This study used the transactional model of stress and coping theory as a conceptual framework, and proposed that hope and coping strategies are sequential mediators for the effects of cyber-victimization on well-being. A total of 337 students aged between 14 and 19 participated in this research (Mage = 16.56). We used the Cyber Victimization Scale, the Forgiveness Scale for Adolescents, the Scale for Coping with Cyber Bullying, and the Well-Being Scale as data collection tools. Pearson Correlation was used to examine the relationships between cyber victimization, coping with cyberbullying, well-being and forgiveness. Afterward, measurement modeling was done using AMOS 22.0 and the PROCESS macro was used for hypothesis testing. The results show that there is a negative relationship between cyber victimization and forgiveness, coping with cyberbullying, and well-being. In addition, forgiveness and coping with cyberbullying was found to have a sequential mediating effect on the relationship between cyber victimization and well-being. The research results provide information on how to increase the well-being of adolescents experiencing cyber victimization.

## Introduction

Cyber victimization is a problem closely related to the mental health and development of adolescents, and interest in studies on cyber victimization has increased in recent years (Bradshaw et al., 2017; Carvalho et al., 2017). Hinduja and Patchin (2008) defined cyber victimization as the targeting of an individual using negative behaviors in a cyber context as a result of adolescent aggression and electronic cooperation. Previous research reveals that cyberbullying and victimization are prevalent in Turkey (Yaman and Sönmez, 2015; Turk and Senyuva, 2021). For example, in Sener et al. (2022) reported the prevalence rate of cyber victimization in adolescents as 35%. Moreover, cyber victimization is considered a major problem in many countries, as the negative consequences of cyber victimization have become a serious public health issue (Zhu et al., 2021). Machimbarrena and Garaigordobil (2018) determined that approximately 8% of adolescents in Spain receive offensive or insulting messages online. Additionally, Chen and Chen (2020) found that in China, Taiwan, and Hong Kong, cyber victimization among adolescents is approximately 30% prevalent.

However, at a critical developmental stage, victimization due to cyberbullying may negatively affect the physical and emotional health of adolescents (Wolke and Lereya, 2015). These include high levels of depression and stress (Martinez-Monteagudo et al., 2020), substance abuse (Sanchez et al., 2017), low self-esteem (Fan et al., 2019), suicidal ideation (Dorol and Mishara, 2021), anger, and shame (Marin-Cortes et al., 2021). Gardella et al. (2017) showed that there are significant relationships between adolescents’ cyber victimization and school attendance problems and academic achievement problems. Extremera et al. (2018) reported that cyber-victim adolescents experienced more negative physical health symptoms and social adjustment problems. Furthermore, a recent study found that higher levels of cyber victimization are associated with increased somatic problems (Strohmeier and Gradinger, 2022).

Clearly, the increasing widespread use of online technology around the world and the popularity of social media platforms among young people can increase the risk of many adolescents becoming cyber victims. More specifically, given that there is a strong correlation between cyber victimization and different emotional problems, it is necessary to find ways to intervene or prevent such problems.

### Relationships between cyber victimization and adolescent well-being

Well-being can be considered as a concept that encompasses all the ways in which people experience and evaluate their lives positively (Wigderson and Lynch, 2013). Tov (2018) defined well-being as a state of long-term satisfaction. (Delgado et al., 2011). However, when relationships among adolescents are characterized by negative experiences such as cyberbullying, it can have negative consequences for well-being.

During adolescence, individuals may be more susceptible to the effects of bullying victimization due to bodily changes, changes in social relationships, and intense emotions. In particular, bodily changes with the onset of adolescence, friendship relationships, individuals’ status within groups at school, and access to peer support are factors associated with cyber victimization experiences (Villora et al., 2020). Studies also show that online peer victimization mostly occurs after negative experiences such as addictive behaviors, emotional distress and interpersonal stress (Cappadocia et al., 2013; Sun et al., 2019). In addition, the responses of cyber bystanders may contribute to the increase of cyberbullying behaviors by supporting the goals of cyberbullies to be dominant, liked, and powerful among their peers (Runions et al., 2013).

In addition, although the high prevalence and negative effects of cyber victimization are well documented, exposure to cyberbullying may not necessarily mean cyber victimization. Individuals experiencing cyber victimization tend to have difficulty coping with their emotions and social relationships (Gamez-Guadix et al., 2015). Moreover, unlike the victimization experienced in traditional bullying, victimization in cyberbullying can develop a negative repetitive effect on the individual as insulting posts remain online and inconvenience the victims for a long time (Schunk et al., 2022). Indeed, research on cyber victimization has revealed that behaviors faced by cyber victims, along with other sources of stress, have potentially negative effects on victim well-being (Zych et al., 2015; Liu et al., 2020).

Although there has been increasing interest in the link between cyber victimization and well-being (Lucas-Molina et al., 2018; Müller et al., 2018) in recent years, the literature on potential variables mediating this relationship is rather limited. Therefore, it seems very important to better understand the coping mechanisms that can buffer the negative effects of cyber victimization on well-being. In this study, we collected data from a sample of Turkish adolescents to examine the mediating effect of forgiveness and coping strategies.

### The mediator role of forgiveness

One of the possible mechanisms for well-being by reducing the effects of cyber victimization can be forgiveness. Forgiveness is the process of changing one’s feelings and attitudes, including anger and revenge, after a negative experience (Barcaccia et al., 2018). Previous studies have revealed that forgiveness has the effect of reducing negative emotions (Roxas et al., 2019; Carpenter et al., 2020). Researchers also found that forgiveness provides justice among individuals, reduces anxiety and depression, and increases well-being (Steiner et al., 2012; Fincham, 2015). Stuntzner et al. (2020) reported that forgiveness as a coping skill is a psychological resource that contributes to the development of psychological well-being. Thompson and Korsgaard (2018) predicted that interventions using forgiveness help reduce negative emotions and maintain well-being in difficult situations.

The possible mediating role of forgiveness on cyber victimization and well-being is consistent with the prediction in the Transactional Theory of Stress and Coping (TTSC) (Lazarus and Folkman, 1984). In this model, forgiveness is an effective emotion-focused coping strategy in terms of reducing the emotion created by the perceived threat and negative emotions related to the victimization (Chan and Wong, 2017). When cyber-victimization occurs, perceiving the situation as stressful may lead some adolescents to deal with it in a vengeful way, such as sending a cyberbullying message to the attacker. In this case, the motivation to forgive can reduce the physiological and psychological problems that adolescents may experience after cyber victimization, and also allow them to increase their general well-being (König et al., 2010).

On the other hand, forgiveness that emerges in cyberbullying experiences should not be seen as a purely internal experience involving reconciliation with the bully. As a matter of fact, forgiving is not condoning wrongdoing, forgetting right away or making excuses (Freedman and Zarifkar, 2016). Rey et al. (2020) showed that a negative nature of forgiveness was associated with depression on cyber victims. For this reason, it is very important to use forgiveness as an emotion-focused and active coping strategy to reduce negative emotions, thoughts and behaviors after a cyberbullying experience. Forgiveness of this nature not only reduces emotional difficulties, but can also revitalize the functioning of well-being by providing a benefit of hope (Van-Rensburg and Raubenheimer, 2015). A functional sense of forgiveness can help break the cycle of future trauma by healing past memories and generating positive responses (Worthington and Aten, 2010). Thus, the current study can provide evidence on how forgiveness is related to the well-being of cyber victims.

### The mediator role of behaviors in coping with cyberbullying

Coping can be defined as an effort to manage stress and related emotions and is very important for maintaining psychological and emotional well-being in the face of negative experiences (Lazarus, 2006). Coping behaviors with cyberbullying also include coping strategies that can be used in the face of cyber victimization. These include using internet safety strategies, parental mediation, confronting the cyberbully, ignoring, blaming oneself or the bully, and obtaining social support from the immediate environment (Trompeter et al., 2018; Heiman et al., 2019; Ngo et al., 2021).

The behaviors of cyber victims to cope with the undesirable consequences arising from their cyber victimization experience may differ. Among the reasons for this difference between individuals are the nature and frequency of the bullying experience, as well as the difference and effectiveness in using coping strategies (Machmutow et al., 2012). In addition, the type of cyberbullying exposed, the level of victimization and anonymity are among the reasons for the difference (Brighi et al., 2019).

Consistent with this approach, Lazarus and Folkman (1987) emphasized that in TTSC, the adequacy of a coping strategy must be evaluated in the context in which it is applied. Accordingly, individuals tend to use problem-focused coping when it is possible to exercise control over the stressful experience and with adequate resources. In contrast, they use emotion-focused coping when they feel that their resources are limited and they can do little to change the situation (Lazarus and Folkman, 1984).

However, examining the methods by which adolescents come out with their cyber-victimization experiences and investigating which coping strategies are positively related to well-being can provide important information about how coping strategies mediate the relationship between cyber-victimization and well-being (Sticca et al., 2015). Studies on coping with cyberbullying and well-being in the literature are quite limited (Schunk et al., 2022). In a previous study, Slonje and Smith (2008) showed that parents and teachers are important sources of information and support for maintaining the well-being of youth victims of cyberbullying. According to Trompeter et al. (2018) reported that problem-focused coping behaviors such as avoiding self-blame and reducing withdrawal from the victim role positively predict well-being. Additionally, some studies have suggested that cyberbullying coping behaviors are mediators for the effects of cyber-victimization on certain psychological outcomes. For example, Jose and Vierling (2018) reported that coping with rumination mediates the effects of cyber-victimization on sleep proficiency, and Zhou et al. (2022) determined that awareness-raising and social support strategies mediate the effects of cyber-victimization on suicidal thoughts. Based on previous research, this study hypothesized that cyber victimization is associated with cyberbullying coping behaviors. Therefore, this study examined whether coping behaviors with cyberbullying mediate the relationship between cyber victimization and well-being.

### Relationships between forgiveness and coping with cyberbullying

In TTSC, it is important to have a protective power such as forgiveness in cyber victimization (Quintana-Orts and Rey, 2018). Forgiveness can help individuals understand how to cope with the consequences of personal harm caused by cyber victimization, as well as protecting adolescents from exposure to cyberbullying. Through forgiveness, victimized youth accept the victimization and can exhibit healthy coping responses by recognizing their thoughts, feelings and behaviors (Mróz and Kaleta, 2022). In addition, the practice of forgiveness in coping with cyberbullying can help the individual to engage in coping behaviors that may reduce the likelihood of engaging in cyberbullying behaviors in the future (Siah et al., 2022). Previous studies have proven strong relationships between forgiveness and coping with bullying behaviors (Ahmed and Braithwaite, 2006; Egan and Todorov, 2009). Lin et al. (2011) found significant correlations between the empathy and ability to recognize the feelings of others and their positive reactions to the bullying experience of students who tend to forgive. Barcaccia et al. (2017) revealed that there is a positive relationship between the forgiveness characteristics of victims and overcoming the negative effects of victimization. In line with the above studies, we predict that forgiveness can predict cyberbullying coping behaviors.

### The sequential mediation effect of forgiveness and coping with cyberbullying

According to the transactional model, encountering a cyberbullying event is a stressful situation (Raskauskas and Huynh, 2015). This theory argues that there is a reciprocal relationship between the stress and coping experienced by the cyberbullying victim. The level of stress experienced by the victim may vary depending on the assessment of difficulties (primary assessment), assessment of options and available resources (secondary assessment), and selected responses (coping) (Lazarus, 1999).

According to TTSC, the cyber victim makes a cognitive appraisal of the negativities in the primary appraisal. Then, the cyber victim focuses on the resources they have to prevent this disturbing situation and initiates a secondary assessment process. If the cyberbully perceives that the difficulty of the negative situation cannot be overcome with the current strategies, the victim may experience stress, and their level of well-being may suffer (Nielsen et al., 2020).

In the model, problem-focused coping includes strategies to solve the problem actively; Emotion-focused coping strategies involve inward-directed emotions from the stressful situation, but limited sharing with others (Tarafdar et al., 2020). Forgiveness, as an emotional strategy in the primary evaluation process, can provide a well-intentioned feeling towards the bully as well as re-establishing the relationship in terms of the trust. Functional forgiveness can repair the damaged relationship and become an essential force for contributing to personal growth. In these aspects, forgiveness differs from other emotional strategies related to forgetting, ignoring, or justifying bullying. Recently, Dans and Muniz (2021) suggested forgiveness as a coping strategy in cyberbullying situations.

As a secondary evaluation, the restorative aspect of forgiving coping may be chosen as a positive coping strategy as it contributes to the cognitive restructuring of the victim individual. The strategy may differ depending on the form of cyberbullying. Teenagers who identify strategies that help reduce the negative effects of cyberbullying situations may develop healthier relationships and feel better in the future (Gonzalez and Fuentes, 2012). Past studies have shown that coping strategies for adolescents can reduce the negative impact of cyberbullying on well-being (Hui and Ho, 2004; Flanagan et al., 2012; Fried, 2013). Consequently, forgiveness and coping behaviors can increase individuals’ efforts to adapt and resist stress as a result of cyberbullying and maintain their well-being.

Within all this information, we assume that this study will contribute to the existing literature by expanding the knowledge on how cyber victimization affects well-being with the sequential mediation model in terms of the TTSC approach. We will also test the impact of forgiveness and coping with cyberbullying behaviors as potential protective factors against cyberbullying. Specifically, we hope that forgiveness and coping with cyberbullying increase the well-being of cyber victims.

### The present study

Although most of the studies on adolescent victims of cyberbullying involve the mental health and well-being of the victims (Lodge and Frydenberg, 2007; Nixon, 2014), there are limited studies on what strategies the victims use when faced with cyberbullying behavior. Previous research has generally focused on the well-being effect of any of the problem or emotion-focused coping strategies in cyber-victimization. Although it is necessary to categorize coping strategies in cyberbullying experiences, using only one of them without problem or emotion-focused coping may fail to define the true coping situation.

Moreover, previous research has found that adolescents commonly use blocking the sender, deleting or avoiding text messages, and reporting to family, teachers, and friends among cyberbullying strategies (Sleglova and Cerna, 2011; Bryce and Fraser, 2013; Slonje et al., 2013). However, there is a lack of evidence in these studies about which strategy works and under what conditions to counter the negative effects of cyberbullying.

Moreover, to our knowledge, there are only a few studies available on forgiveness and coping strategies as possible mediators of the effects of cyber victimization on well-being (Sanchez-Hernandez et al., 2021; Quintana-Orts et al., 2022). This research used TTSC to examine the effects of cyber victimization on well-being. It is estimated that cyber victims may evaluate the act of cyberbullying as a threat and may lead to the adoption of forgiveness and different coping strategies based on their secondary evaluations of the options for coping with cyber victimization.

On the other hand, unlike other studies, this study used forgiveness as a functional coping method with other adaptive strategies between cyber victimization and well-being. This suggests that in cyberbullying, interventions may be needed more to develop forgiveness skills and to respond to cyber victimization more adaptively.

In addition, conducting this study at a time when COVID-19 is spreading may provide deeper insights into the well-being of adolescents. When the Covid-19 disease emerged, states took various measures to prevent the spread of the disease. Among the most striking measures are the closure of schools and the introduction of curfews. Experts aimed to minimize the level of social contact of people with these measures. However, these measures reduced the spread of the disease and negatively affected the well-being of adolescents (Danese et al., 2020; Jessica et al., 2021). For example, Jackson et al. (2021) revealed that the level of well-being of adolescents decreased during the pandemic period. During the Covid-19 pandemic, limiting the daily lives of adolescents made them use more social media tools (Leung and Shek, 2020; Peker et al., 2021). Therefore, this study can also provide new information about the relationships between cyber victimization and well-being in this process where intensive technological tools are used.

Based on the above theoretical explanations, the present study aimed to analyze how forgiveness and cyberbullying coping behaviors work together as two mediators in a series of mediation models in the Turkish adolescent sample.

**Figure 1:**
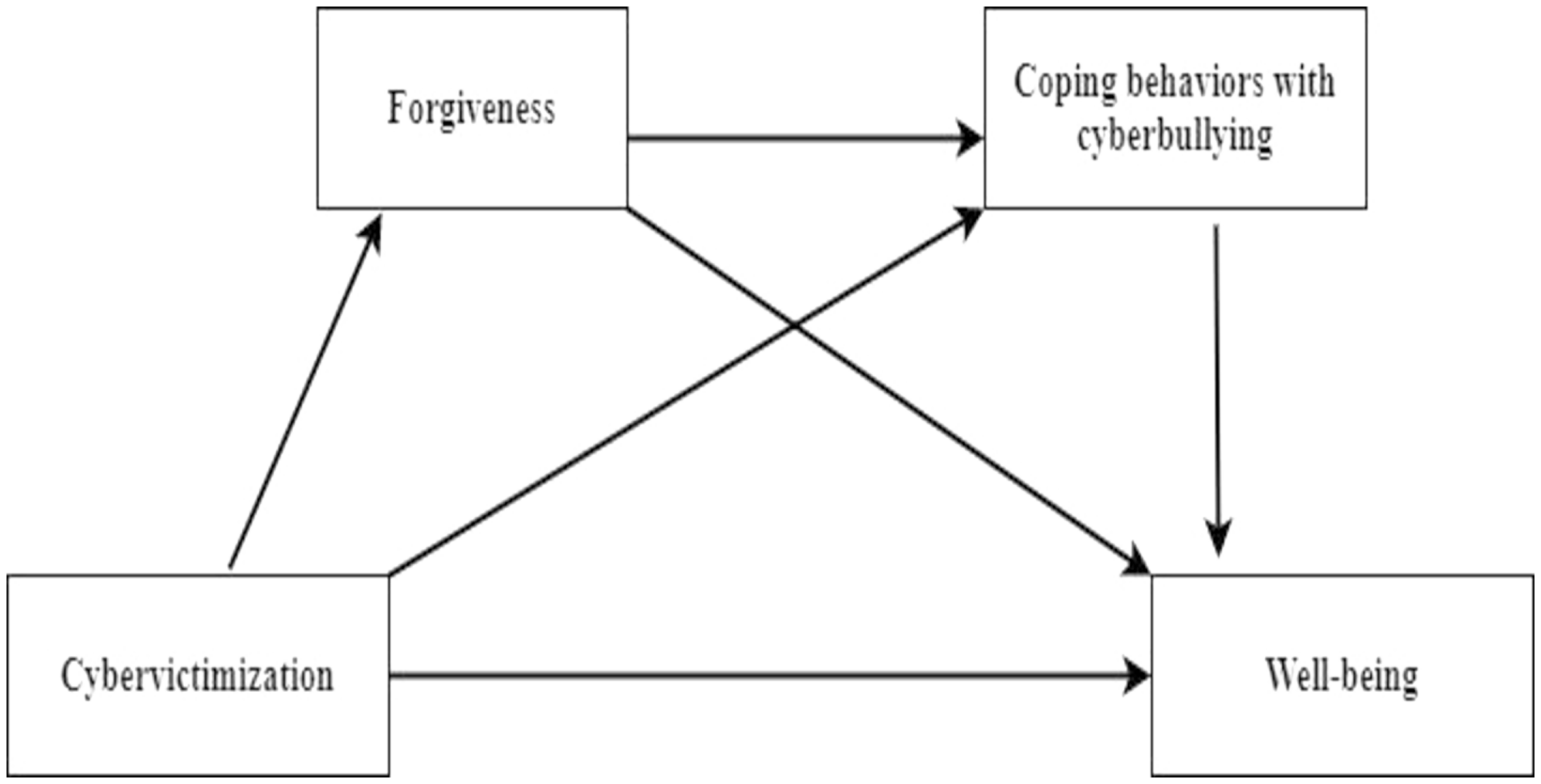
The serial multiple mediation of forgiveness and coping behaviors with cyberbullying in cyber victimization and well-being.

Compared to a simple mediation model, the serial multiple mediation model (Figure 1) allows simultaneous examination of multiple variables from the antecedent to the next variable in a single model, which can offer more insight into how cyber victimization is related to well-being (Quintana-Orts et al., 2020). Therefore, this model has an important feature in improving the theory by addressing the underlying mechanisms, contributing to interventions and preventing the decline in well-being after experiencing cyber victimization.

In the serial mediation model in Figure 1, cyber victimization is expected to predict well-being. In the model, it is predicted that the relationship between cyber victimization and well-being will be mediated by the behaviors of forgiveness and coping with cyberbullying. However, we hypothesized that mediators (forgiveness and cyberbullying coping behaviors) have a direct impact on each other. Finally, in the model, we assumed that forgiveness and coping with cyberbullying would act as a serial mediator between cyber victimization and well-being. Therefore, in this study, based on the TMS model, we suggested that the well-being levels of adolescents exposed to cyber victimization decreased indirectly, especially in a sequential manner, more cyber victimization was associated with lower forgiveness and coping behaviors, and these were associated with lower well-being. Consistent with the proposal, we formed our hypotheses as follows:

*H1*: Cyber victimization is negatively related to well-being.

*H2*: Forgiveness mediates the relationship between cyber victimization and well-being.

*H3*: Coping behaviors with cyberbullying mediate the relationship between cyber victimization and well-being.

*H4*: Forgiveness is positively related to cyberbullying coping behaviors.

*H5*: Forgiveness and cyberbullying coping behaviors sequentially mediate the relationship between cyber victimization and well-being.

## Materials and methods

### Participants

We created some criteria to identify the participants. (I) Living in Erzurum, Turkey, (II) Participants aged between 14 and 19 years old, (III) Reading and understanding Turkish, (IV) using at least one of the social networking sites in the last 6 months (Instagram, Twitter, Facebook, etc.) (V) To obtain informed consent form approval from the parents of the students who will participate in the application.

The population of the research consists of 12.500 students studying in 46 high schools in Erzurum in December of the 2021 academic year. At the time of the study, the Covid-19 epidemic disease was ongoing. In order to prevent the spread of the epidemic, the Ministry of National Education took measures such as wearing masks in schools, having a certain number of students in the classrooms, shortening the duration of the lessons, communicating with their friends by keeping a certain distance, and conducting some of the lessons online. However, the rate of going to school was low because the students were worried about both contracting the epidemic and transmitting it to their families and close circles. Taking into account the low rate of student enrollment, the researchers identified 16 schools with 750 or more students in order to reach more students. We used the proportional stratification sampling method to determine which of these schools to apply the scales to. Accordingly, we calculated the percentage distribution of 16 schools according to their types. 50% of the schools were Anatolian High School (*n* = 8); 37% are Vocational High School (*n* = 6), 13% are Anatolian Imam Hatip High School (*n* = 2). At the last stage, considering the percentage distribution of 16 school types, we decided to include 4 schools from Anatolian High School, 2 schools from Vocational High School and 2 schools from Anatolian Imam Hatip High School.

The G*Power 3.1 program was used to determine the sample size and power. Results a minimum of 352 samples were reached for models with an alpha level of 0.05 and power level of.80. This many participants can be considered sufficient for the sample size in the current study (Faul et al., 2007). We did not include the data of 7 students who did not meet the normality criteria and 8 students who filled in the scales incompletely. As a result, we analyzed the data set of 337 students. In the current study, 50.1% (*n* = 169) of the students are boys and 49.9% (*n* = 168) are girls. In addition, 17.5% of the students are in the 9th grade, 21% are in the 10th grade, 16% are in the 11th grade and 45% are in the 12th grade.

### Procedure

The researchers received the necessary approval from the Erzurum Provincial Directorate of National Education for the study to be implemented in schools. After the schools had been determined to be implemented, the researchers visited the guidance services in these schools. The researchers explained the purpose of the study to the psychological counselors working in the guidance service. Then, the researchers asked the psychological counselors to apply the prepared scales to the students who were exposed to cyberbullying and applied to the guidance service, by the voluntary principle. The researchers left the prepared scales and the informed consent form for the consent of the parents to the guidance service. The data collection process started after ethical approval was obtained and we completed it in approximately 25 days (11.11.2021). The data were collected by psychological counselors working in the school guidance service during school hours. Students answered the scales in approximately 20 min. In this study, 28 students who stated that they did not want to participate voluntarily and 17 students who did not receive parental consent were not included.

### Measures

#### Revised cyberbullying inventory

We used the Revised Cyberbullying Inventory (RCI) to determine the cyber victimization status of adolescents. Topcu and Erdur-Baker (2010) developed the inventory. The inventory consists of two separate parts, cyberbullying and cyber victimization, and consists of 28 items (Sample items; “threatening in the chat room,” “mocking the comments on a forum site,” and “deceiving the other party by showing the gender differently”). The cyberbullying and cyber victimization behaviors of the participants are evaluated on a 4-point Likert level (never, once, twice, and three times, more than three). In this study, we used the Cyber Victimization dimension (Example articles; threatening in the chat room, mocking the comments and information on a forum site, deceiving the other party by showing the gender differently). The internal consistency reliability coefficient of the Cyber Victimization Inventory is 0.75. In this study, we performed AFC to calculate the validity of the Cyber Victimization Inventory (RCVI). AFC results confirm the single-factor structure of RCVI (*χ*2*/*sd = 2.27; NFI = 0.97; CFI = 0.98; RFI = 0.97; GFI = 0.90; SRMR = 0.041; RMSEA = 0.047). The internal consistency reliability coefficient of the Cyber Victimization Inventory for this study was.78.

#### The five-dimensional scale of well-being for adolescents (EPOCH)

We used the scale developed by Kern et al. (2015) to determine adolescents’ well-being levels. Demirci and Eksi (2015) adapted the scale to Turkish. The scale consists of 20 questions in total. Items on the scale have a 5-point Likert rating between 1 (never) and 5 (always). The scale has dimensions of commitment, determination, optimism, connectedness, and happiness (Sample items; I am completely immersed in what I do-commitment dimension-; I finish what I start-determination dimension-; I am optimistic about my future-optimism dimension-; I have friends that I care about-relatedness dimension-; I feel happy-happiness dimension-). The internal consistency coefficient of the total score of the scale was 0.95; sub-dimensions vary between.72 and.88. The well-being scale can be evaluated by taking a total score. In this study, we performed AFC to calculate EPOCH validity. AFC results show that the five-factor structure of EPOCH is confirmed (χ2*/*sd = 2.95; NFI = 0.94; CFI = 0.93; RFI = 0.96; GFI = 0.90; SRMR = 0.050; RMSEA = 0.053). In this study, the overall internal consistency coefficient of EPOCH was.88.

#### Forgiveness scale for adolescents

The Forgiveness Scale was developed by Asıcı and Karaca (2018). The scale consists of 21 questions in total. The items on the scale have a 5-point Likert rating from 1 (does not describe me at all) to 5 (describes me completely). The scale has four sub-dimensions: “components of forgiveness,” “maintaining anger,” “revenge” and “empathy” (Sample items; I am tolerant of people who have wronged me -components of forgiveness dimension-; I dream of taking revenge on the person who has wronged me -revenge dimension-; I’m prone to grudges- maintaining anger -; I try to understand the feelings of the person who mistreated me-empathy-). The overall Cronbach’s alpha internal consistency coefficient of the scale is 0.90 and respectively, the sub-dimensions are.86, 0.76, 0.85, and.75. The Forgiveness Scale can be evaluated by taking a total score. In this study, we performed AFC to calculate the validity of the “Forgiveness Scale for Adolescents (FSA).” AFC results show that the four-factor structure of the FSA is confirmed (*χ*2*/*sd = 3.28; NFI = 0.92; CFI = 0.94; RFI = 0.93; GFI = 0.90; SRMR = 0.058; RMSEA = 0.058). The FSA total score internal consistency reliability coefficient for this study was 0.85.

#### Scale for coping with adolescents’ cyberbullying (SCAC)

The Adolescents Scale for Coping with Cyberbullying was developed by Peker et al. (2015). The scale consists of 17 items. The scale has 4 sub-dimensions: seeking social support, seeking help, coping, and online safety. In addition, the scale has a 4-point Likert-type rating as “Never (1), Sometimes (2), Usually (3), Always (4)” (Sample items; I talk to someone I trust about what’s going on -social support-seeking dimension-, I consult my teacher about what I can do-help-seeking dimension-, I want people who act like this to stop this behavior-fight dimension-, I do not share passwords of my accounts with others -online security dimension-). The second level confirmatory factor analysis results show that the scale can be used as a single factor (*x*^2^/sd = 3.28; NFI = 0.92; CFI = 0.94; RFI = 0.90; GFI = 0.91; SRMR = 0.069; RMSEA = 0.072; Peker et al., 2020). The total internal consistency reliability coefficient of the scale is 0.83. The internal consistency reliability coefficients of the scale ranged from.70 to.86. High scores obtained from the scale show that the behavior of coping with cyberbullying is also high. The scale for coping with cyberbullying can also be used by taking the total score for adolescents. In this study, we performed AFC to calculate the validity of the Scale for Coping with Adolescents’ Cyberbullying. AFC results show that the four-factor structure of the USSR is confirmed (*χ*2*/*sd = 3.58; NFI = 0.91; CFI = 0.92; RFI = 0.91; GFI = 0.90; SRMR = 0.060; RMSEA = 0.068). The overall internal consistency reliability coefficient of the scale for this study is.80.

### Data analysis

Researchers calculated the arithmetic mean, standard deviation, and Pearson correlation coefficients of participants’ cyber victimization, forgiveness, coping with cyberbullying and well-being using the SPSS 22.0 program. Researchers conducted a structural equation model analysis to examine the mediating role of forgiveness and coping with cyberbullying with the AMOS program. We used the Maximum Likelihood method in the analysis. First, we examined the direct impact of cyber victims on well-being. Second, we examined the predictive level of forgiveness and coping with cyberbullying between cyber victimization and well-being. In the third stage, we added the variables of forgiveness and coping with cyberbullying as mediators to the relationship between cyber victimization and well-being.

We adopted some indices to evaluate model fit. According to the model fit indices, χ2/sd being less than 5, GFI, CFI, NFI greater than 0.90, and RMSEA and SRMR values less than.08 indicate an acceptable model fit (Kline, 2015). We also performed a Bootstrap analysis, which is frequently used in mediation approaches, to determine the significance level of indirect effects in the research. In this context, we used the PROCESS macro program of Hayes (2013) (Model-6). For this, we chose the 5,000 sampling method and made sure that the confidence intervals did not contain zero (Preacher and Hayes, 2004).

### Ethics statement

Ethics approval was obtained from Atatürk University Educational Sciences Ethics Committee before conducting the survey. (Date: 11.11.2021, meeting number: 12 and decision number: 23).

## Results

### Relationships between cyber victimization, forgiveness, coping with cyberbullying, and well-being and descriptive results

We used Pearson correlation analysis to reveal the relationship between students’ cyber victimization, forgiveness, coping with cyberbullying, and well-being scores. In addition, we performed descriptive statistical operations on the variables. Descriptive and correlation results for the variables are presented in Supplementary Tables 1, 2.

Supplementary Table 1 shows the arithmetic mean and standard deviation scores of the variables. However, the skewness and kurtosis values of the research variables are between 2 and − 2. This shows that the data meet the normality assumption (Tabachnick and Fidell, 2012).

Supplementary Table 2 shows that there are low-level, negative-significant relationships between well-being and cyber-victimization (*r* = 0.23; *p < 0*.05). It is observed that there is a moderate, positive, and significant relationship between well-being, and forgiveness (*r* = 0.32; *p < 0*.05). It was found that there is a moderate, positive, and significant relationship between well-being, and coping with cyberbullying (*r* = 0.37; *p < 0*.05).

There is no multicollinearity problem as the relationships between the variables are not over.90. We examined the covariance of the data before examining its multiple normalities. We observed that the values in the P–P plot are on a line, and in the scatter plot, the data are around a line. To test the normality of the regression errors, we examined the skewness and kurtosis values of the residuals. As a result of these analyzes, we determined that the regression residual values were close to normal (Tabachnick and Fidell, 2012). Since it was observed that the skewness value for the normality of the regression errors was −0.091 and the kurtosis value was −0.68, we concluded that the regression residual values showed a distribution close to the normal. Researchers used the Mardia Test to determine the assumption of multivariate normality of the data. As a result of the analysis, we observed that the multivariate normality assumption was confirmed (*p* > 0.05.).

### Mediation model

#### Measuring model

Researchers established a measurement model with 4 latent variables (cyber victimization, forgiveness, coping with cyberbullying, and well-being) and 27 observed variables. We determined that the established measurement model was a good fit [GFI = 0.90, CFI = 0.91; NFI = 0.92; SRMR = 0.050; *χ*2/sd = 2.74; RMSEA = 0.074]. In addition, the researchers found that all observed variables significantly loaded on their respective latent structures (*p* < 0.05 between.35 and.78).

#### Structural model analysis

To test the hypotheses, we first examined the direct pattern between cyber victimization and well-being. The results show that cyber victimization has a positive, negative and significant effect on well-being (*β* = −0.22; *p* < 0.01.; *t* = −3.32; GFI = 0.90; CFI = 0.91; NFI =0.91; SRMR = 0.070; *χ*2/df = 3.09; RMSEA = 0.079). Then, we added the variables of forgiveness and coping with cyberbullying as mediators between cyber victimization and well-being. We observed that when both variables were added to the model, they predicted the relationship between cyber victimization and well-being at a significant level (CV-FRG: *β* = −0.35; *p* < 0.01.; *t* = −3.19; FRG-CBCW: *β* = 0.18; *p* < 0.01.; *t* = 2.04; CBCW-WB: *β* = 0.32; *p* < 0.01.; *t* = 4.44; GFI = 0.92; CFI = 0.90; NFI = 0.93; SRMR = 0.068; *χ*2/df = 2.69; RMSEA = 0.071). Finally, we examined the multi-order mediation relationship between cyber victimization and well-being. We determined that there were significant decreases in the relationship between cyber victimization and well-being, according to the first model, which examined the mediation relationship between forgiveness and coping with cyberbullying (*β* = −0.06; *p* < 0.01; *t* = −1.00; GFI = 0.93; CFI = 0.92; NFI = 0.92; SRMR = 0.060; *χ*2/df = 2.70; RMSEA = 0.071).

Considering all fit index values, it shows complete mediating role of forgiveness and coping with cyberbullying in the relationship between cyber victimization and well-being. Cyber victimization, forgiveness, and coping with cyberbullying explain 23% of well-being. The estimated and standardized values of the variables are shown in Supplementary Table 3.

**Figure 2:**
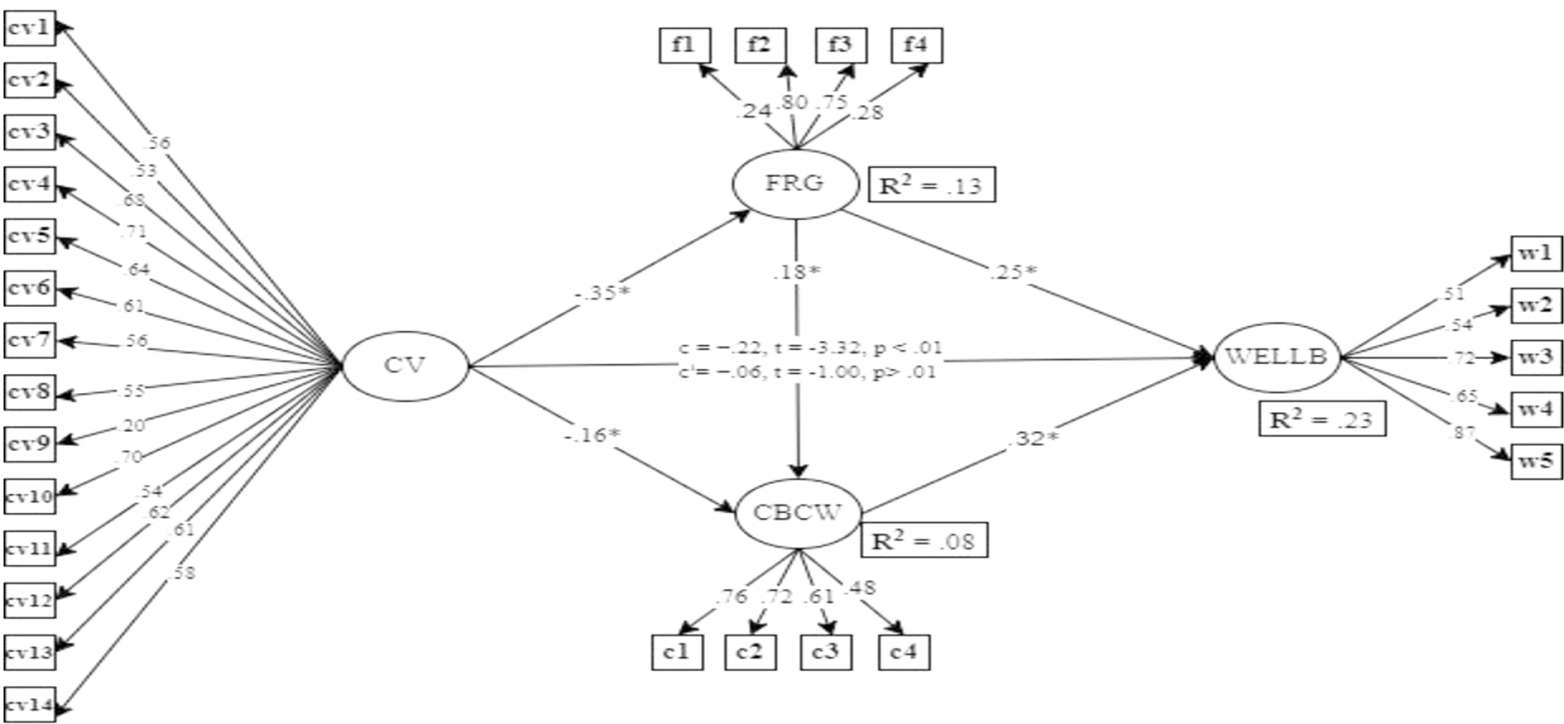
The mediating role of forgiveness and coping with cyberbullying between cybber victimization and well-being. CV, Cyber victimization; CBCW, Coping with cyberbullying; FRG, Forgiveness; WB, Well-being. *p < 0.05.

Figure 2 shows the standardized values of forgiveness and coping with cyberbullying between cyber victimization and well-being.

#### Bootstrapping process

We used the bootstrap method to determine the direct and indirect effects on the structural model. The coefficients for the direct and indirect effects resulting from this process and the confidence intervals for these coefficients are shown in Supplementary Table 4.

Supplementary Table 4 shows that direct paths are significant and cyber victimization indirectly affects well-being.

## Discussion

### Cyber victimization well-being prediction

Findings regarding the first hypothesis of the study reveal that cyber victimization negatively predicts well-being. The results confirm Hypothesis 1. Findings show that exposure to cyberbullying reduces well-being. This result is consistent with previous research results that found cyber victims to have lower well-being (Moore et al., 2012; Palmeri, 2013; Przybylski and Bowes, 2017; Reignier et al., 2022).

The level of well-being of cyber victims may decrease compared to those who have not experienced bullying (Navarro et al., 2015). Cyber victims may have few internal resources and poor self-regulation skills to deal with this negative situation (Mazzone et al., 2017). Cyber victims may experience feelings of worthlessness, rejection, and helplessness (Peker, 2017). The results for the first hypothesis reveal the negative effect of cyber victimization on well-being.

### The mediating role of forgiveness in the relationship between cyber victimization and well-being

Findings regarding the second hypothesis of the study reveal that forgiveness has a mediating effect on the relationship between cyber victimization and well-being. This result confirms Hypothesis 2. The results of the research are consistent with the results of previous studies showing that there is a relationship between the forgiveness of victims and well-being (Skaar et al., 2016; Quintana-Orts and Rey, 2018).

Forgiveness can encourage the victim not to engage in aggressive behavior towards the other person (Watson et al., 2015). Akhtar and Barlow (2017) stated that forgiveness is a protective factor to reduce violent behavior and improve mental health. Hui et al. (2011) state that forgiveness contributes to the increase of well-being by reducing the negative feelings of the victims such as hurt, sadness, anger, and revenge. Peets et al. (2013) revealed that forgiveness affects individuals’ well-being positively by reducing aggressive behaviors.

Since the forgiveness process is primarily about internalized reactions to experiences that may negatively affect it, it can be considered an emotion-focused active coping style (Strelan and Covic, 2006). Forgiveness is an emotional strategy that can help you discuss what happened with the cyberbully and seek compensation, stop the bullying experience, and figure out what to do next. Forgiveness-focused emotional intervention can reduce the salience of perceived threats by regulating emotions in experiences where problem-focused strategies are ineffective (Worthington and Scherer, 2004).

The cyber victim may realize that as a result of their forgiveness behavior, they may encounter a situation in which they also need forgiveness. In addition, a functional sense of forgiveness may contribute to the individual taking precautions before encountering the same problem again. Active forgiveness can make the cyber-victim realize that a past abuse they have suffered is an opportunity to learn more about themselves (Gordon and Baucom, 1998). Therefore, a person who is forgiving in any of the experiences in the context of cyberbullying can be expected to have proactive or preventive coping skills as a way of preserving valuable relationships.

### The mediating role of coping with cyberbullying in the relationship between cyber victimization and well-being

Findings regarding the third hypothesis of the study reveal that coping with cyberbullying has a mediating effect on the relationship between cyber victimization and well-being. This result confirms Hypothesis 3.

Lazarus and Folkman (1987) report that if the used coping strategies have a positive effect on well-being, there is a high probability of using the same strategies in similar situations in the future. In the context of cyberbullying, this approach is supported by previous researchers (Riebel et al., 2009; Perren et al., 2012). Perren et al. (2012) state that strategies such as reacting directly to cyberbullying acts of cyber victims, ignoring them, seeking social support, and using technological solutions can be effective in increasing well-being. In a similar study, Machackova et al. (2013) revealed that cyber victims’ use of strategies such as sharing information, hiding evidence, and blocking the account to prevent access to the bully increased their well-being.

Common responses to coping with cyberbullies recently include ignoring cyberbullying behaviors, telling parents or teachers, and student commitment (Bradshaw et al., 2014). A sense of belonging among peers and students’ willingness to help each other can promote healthy well-being and prevent problem behaviors (Sleglova and Cerna, 2011). Cyber victims can feel better by using technical coping strategies such as hiding and blocking online comments (Byrne, 2021). However, cyber victims can also resort to online combating strategies to stop the bully (Peker et al., 2015).

Cyberbullying can occur at any time and can have multiple effects on the victim in a short time. Especially when young people perceive the effects of cyberbullying, they are more exposed to the negative consequences of cyberbullying behaviors because they lack the necessary knowledge and resources to cope. Therefore, adolescents need to be more informed about appropriate coping strategies against cyberbullying.

### Relationships between forgiveness and coping with cyberbullying

Findings regarding the fourth hypothesis of the study reveal that forgiveness positively predicts coping with cyberbullying. This result confirms Hypothesis 4. Forgiveness in the context of bullying has been associated with positive coping strategies such as conflict resolution and support seeking, higher self-esteem, and lower levels of social anxiety (Flanagan et al., 2012). The cyber victim begins to develop themselves and adapt to the environment thanks to forgiveness. Thus, they can resist bullying behaviors and apply appropriate strategies in later life. For example, Ogurlu and Sarıcam (2018) report that forgiveness has positive effects on determining effective mechanisms in coping with bullying. Perez (2008) emphasizes that forgiveness may be closely related to strategies that include internalizing behaviors in the face of bullying. Therefore, forgiveness can increase the sense of personal empowerment and interpersonal power, and enable adolescents to use functional strategies in bullying experiences.

### The sequential mediation effect of forgiveness and coping with cyberbullying

Findings related to the fifth hypothesis of the study reveal that forgiveness and coping behaviors with cyberbullying have a sequential mediating role between cyber victimization and well-being. This result confirms Hypothesis 5. According to this finding the research, cyber victimization predicts forgiveness, forgiveness predicts cyberbullying coping behaviors, and cyberbullying coping behaviors predict well-being at a significant level. Findings on the sequential mediation role obtained at the end of the study show that the operational model of coping with cyberbullying is confirmed.

Forgiveness is associated with positive gains such as positive emotional regulation and adaptive responses to stress (Wade et al., 2014). Liu et al. (2013) state that forgiveness can potentially protect negative thoughts and strategy choices that cyber victims may experience. Carlon et al. (2014) reported that increasing self-forgiveness will enable bullied students to realize their behavior and stay away from unhealthy coping.

In addition, the use and effectiveness of coping strategies can affect individuals’ well-being (Singh and Bussey, 2009; Sticca and Perren, 2013). These strategies are very important for maintaining emotional and psychological well-being in cyberbullying experiences, as in many negative situations. Consistent with the model of this research, Lazarus (2006) argues that a particular coping style should not be evaluated without considering the current situation. Therefore, since problem-focused and emotion-focused coping complement each other in most stressful situations, they should not be seen as two different types of coping.

Some previous studies show that blocking, deleting bad messages, and stopping internet use can be beneficial actions for the well-being of cyber victims (Price and Dalgleish, 2010; Livingstone et al., 2011). Skrzypiec et al. (2012) emphasize that high-quality friendship and perceived support can be a protective factor against negatively affecting well-being among bullied children. Banerjee et al. (2010) state that watching websites and providing emotional support can have positive effects on the well-being of cyber victims.

As a result, it can be predicted that adolescents who can cope with a negative experience online and exhibit functional strategies to protect themselves from possible harm have higher well-being levels.

### Practical implications

Our results expand the knowledge in the literature on how to protect the well-being of cyber victims. In addition, there are several practical implications associated with our findings. First, this study revealed that forgiveness positively affects the well-being of cyber victims. Therefore, increasing forgiveness can be one of the important ways to increase well-being. Psycho-educational programs can help increase forgiveness, which can increase well-being. In particular, the presence of content on emotions such as revenge, anger, sadness, and guilt in these programs can contribute to the well-being of cyber victims. Secondly, this study revealed that coping behaviors with cyberbullying positively affect the well-being of cyber victims. In this regard, improving cyberbullying coping behaviors can help increase the well-being of cyber victims. Awareness work by mental health professionals in schools can help increase the coping behaviors of cyber victims, which can increase well-being. In particular, awareness activities may include information such as asking for help from family and friends when individuals encounter a negative situation while using information and communication technologies, not sharing their passwords related to social networking sites with others, and not opening messages from people they do not know and taking online security measures. Moreover, mental health professionals can also train students in active coping skills, such as warning the other person when they encounter cyber bullying, and emphasizing that what they are doing is wrong. These studies by mental health experts can ensure that individuals’ well-being levels are maintained in case of cyber victimization.

### Limitations and future direction

Although there are theoretical and practical implications, this research has some limitations. First, although this study presents several prospective findings, there are limitations to its generalizability. The sample of the study consists of a limited number of adolescent individuals living in a city in Turkey. Therefore, the individuals participating in the study do not fully represent the Turkish adolescent population. In this sense, using a more balanced sample size from different regions to validate and generalize our results may yield interesting results. A second limitation of this study is the small sample size. Since the study was carried out during the pandemic period, there were frequent restrictions. Therefore, the researchers had difficulties finding individuals to participate in the study. After the COVID-19 quarantine, a wider audience can be reached, as there will be easier mobility among students and more intense participation in academic activities. Third, the use of a cross-sectional design to explain the inter-variable cause-effect situation in this study may prevent us from concluding the direction of the effect. Therefore, we must be careful in making causal inferences. Fourth, the scales used for the mediator variables in the study do not cover all the features of the variables. For example, the scale of forgiveness we used in this study consists of 3 sub-dimensions. Therefore, scales containing different factors can be used to understand the forgiveness process of adolescents. In future studies, scales involving other factors such as forgiving oneself and others, forgiving the situation, and focusing on the positive can be used in future studies. Again, a scale including emotion-focused coping strategies can be used in coping with cyberbullying. Finally, adolescents with culturally similar structures participated in this study. Researchers can repeat the model in adolescents with different socio-cultural elements in Turkish culture. However, immigrant individuals living in Turkey were not included in the study. In future studies, the relationships between cyber victimization and well-being of immigrant adolescents can be examined.

## Conclusion

This study has revealed that forgiveness and coping with cyberbullying are serial mediators between cyber victimization and well-being in the Turkish adolescent sample. Therefore, forgiveness and coping with cyberbullying among adolescents experiencing cyber victimization should be seen as a promising way to improve well-being. In addition, our findings not only expand the knowledge of the literature on how to increase the well-being of cyber victims but also offer mental health professionals a new perspective on maintaining the well-being of individuals who encounter cyberbullying behaviors. Moreover, the possible serial mediator role of forgiveness and coping with cyberbullying on cyber-victimization and well-being is consistent with TTSC, and our findings provide deeper insights into the model.

## Data availability statement

The original contributions presented in the study are included in the article/Supplementary material, further inquiries can be directed to the corresponding author.

## Ethics statement

The studies involving human participants were reviewed and approved by Atatürk University Educational Sciences Ethics Committee Unit. Written informed consent to participate in this study was provided by the participants’ legal guardian/next of kin. Written informed consent was obtained from the individual(s), and minor(s)’ legal guardian/next of kin, for the publication of any potentially identifiable images or data included in this article.

## Author contributions

YE, AP, and SC determined the research topic together and wrote the discussion part of the article. YE wrote the introduction part. SC wrote the method part. AP wrote the findings section. All authors contributed to the article and approved the submitted version.

## Conflict of interest

The authors declare that the research was conducted in the absence of any commercial or financial relationships that could be construed as a potential conflict of interest.

## Publisher’s note

All claims expressed in this article are solely those of the authors and do not necessarily represent those of their affiliated organizations, or those of the publisher, the editors and the reviewers. Any product that may be evaluated in this article, or claim that may be made by its manufacturer, is not guaranteed or endorsed by the publisher.

## Supplementary material

The Supplementary material for this article can be found online at: https://www.frontiersin.org/articles/10.3389/fpsyg.2022.819049/full#supplementary-material
