# Supplementary figures and images for: Cyber victimization and well-being in adolescents: The sequential mediation role of forgiveness and coping with cyberbullying
Source: Front Psychol. 2022 Nov 18;13:819049. doi: 10.3389/fpsyg.2022.819049 (PMC9716218; doi:10.3389/fpsyg.2022.819049)

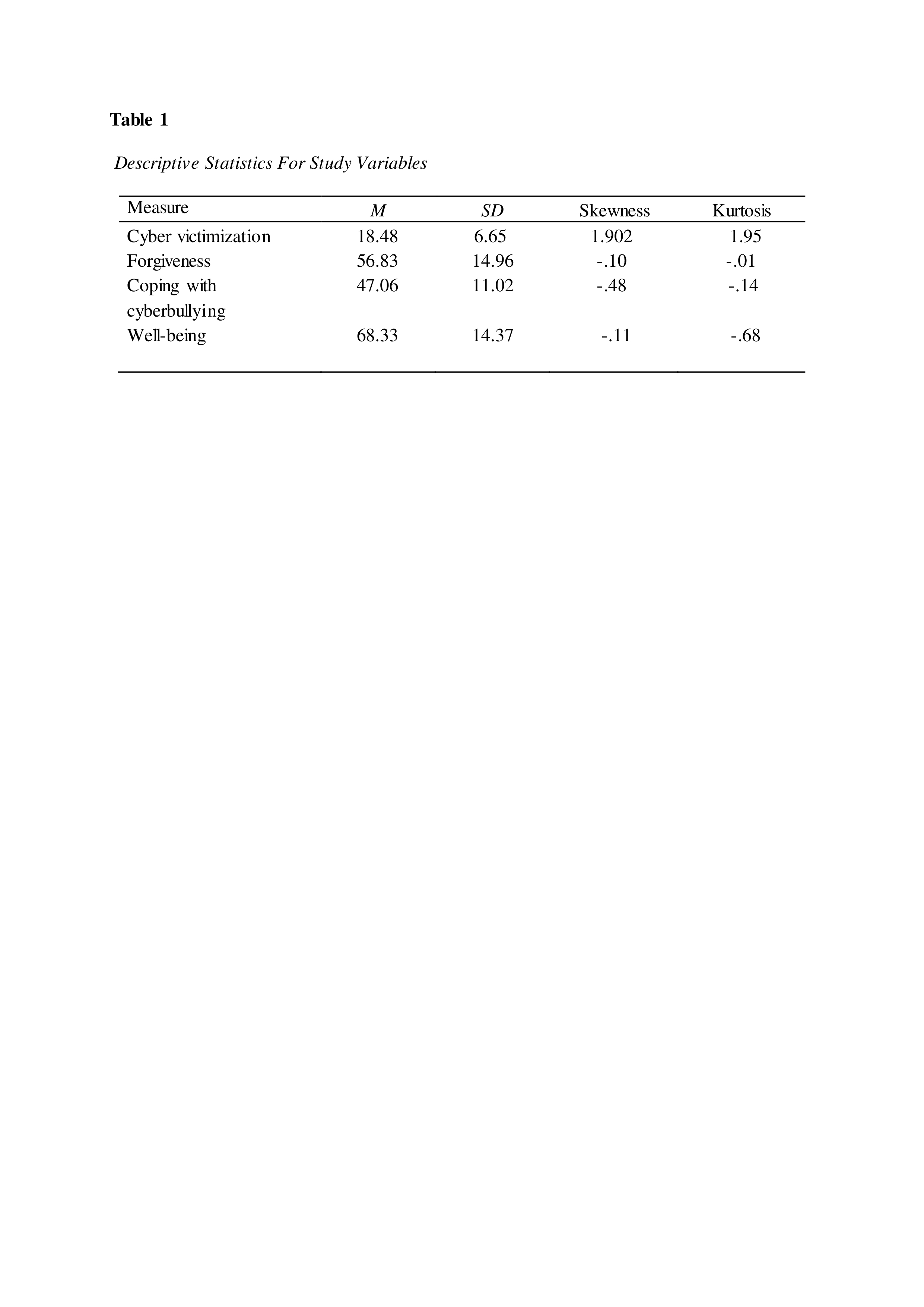

Supplement: Supplementary file 1 [file Image_1.JPEG]

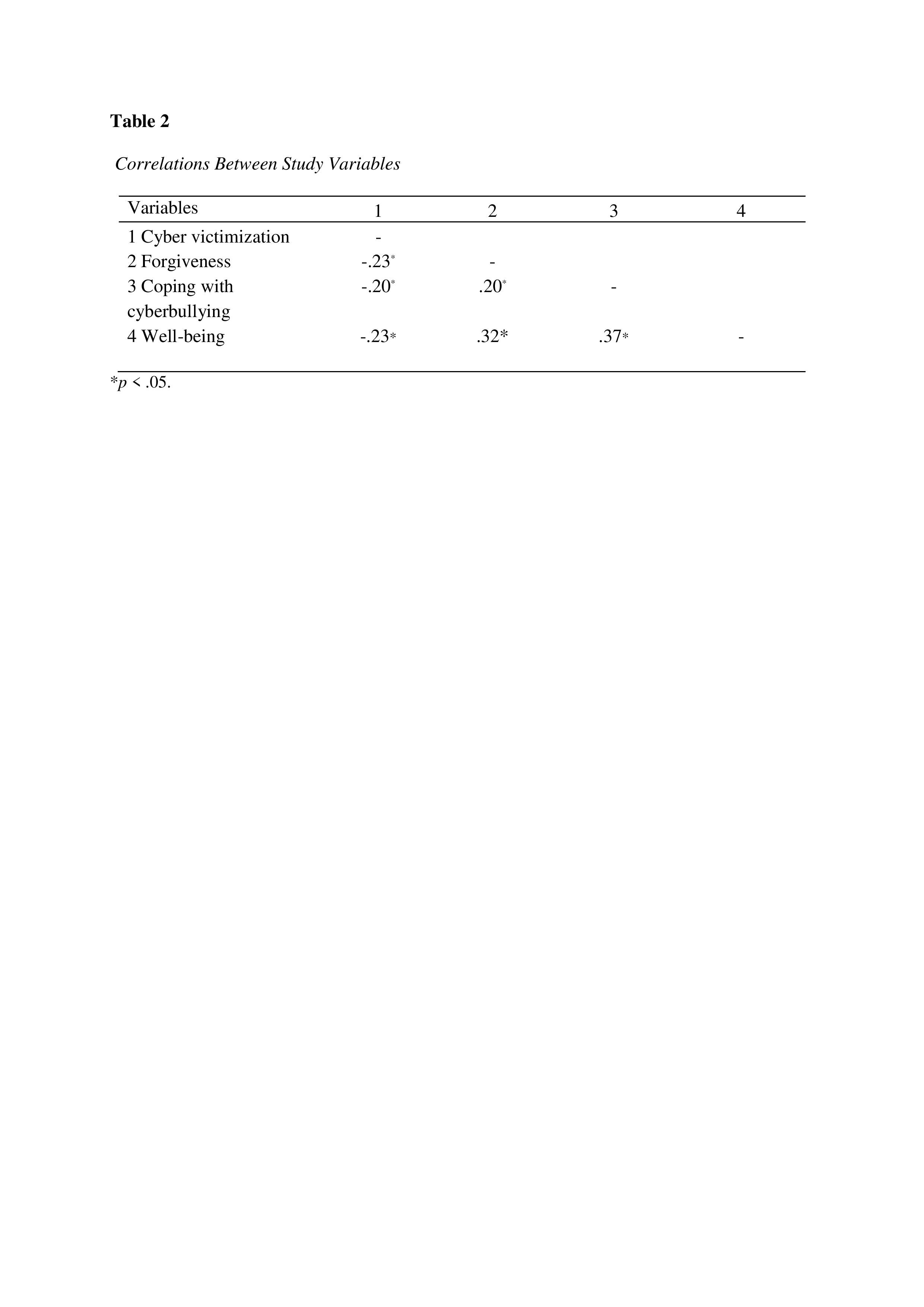

Supplement: Supplementary file 2 [file Image_2.JPEG]

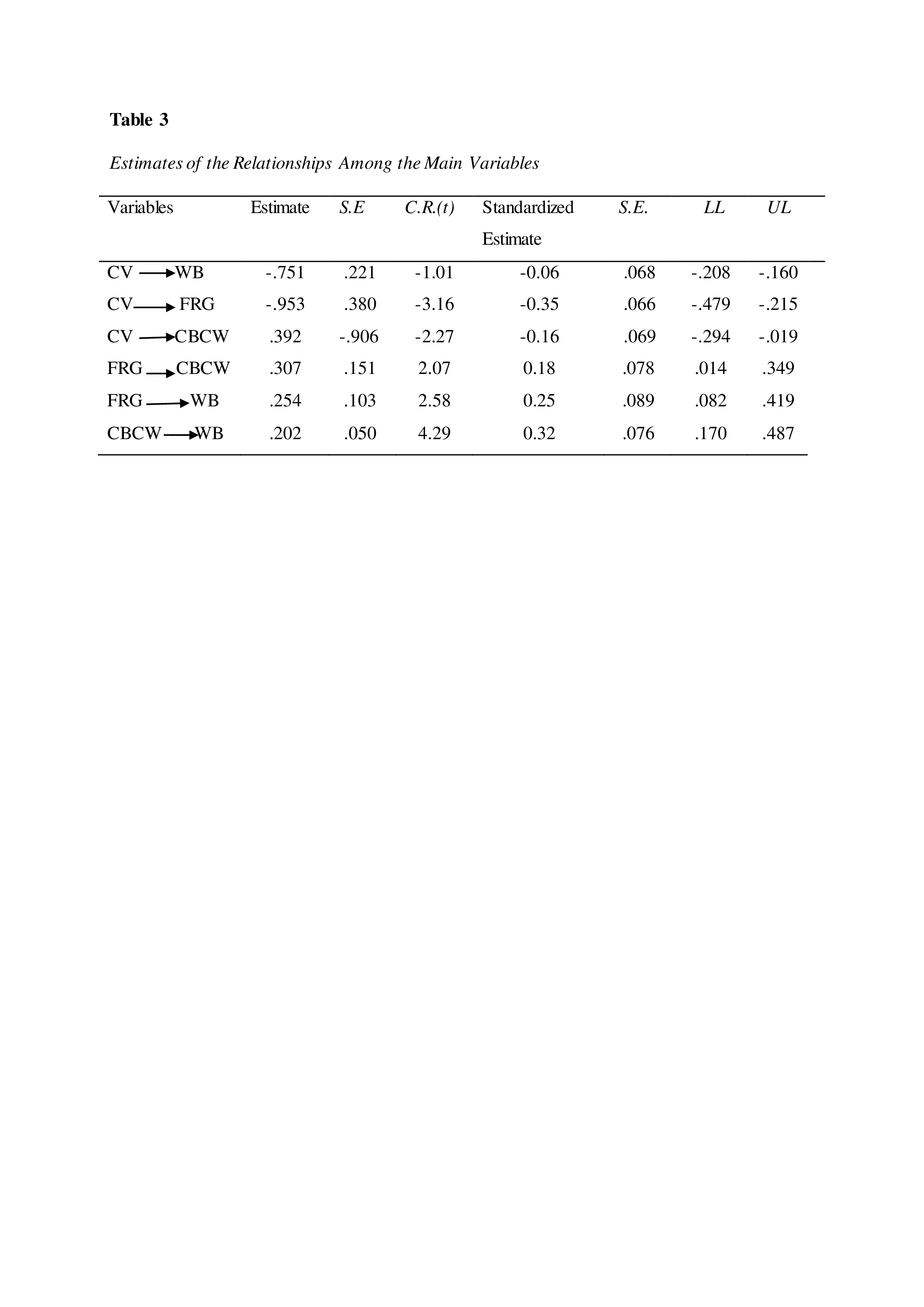

Supplement: Supplementary file 3 [file Image_3.JPEG]

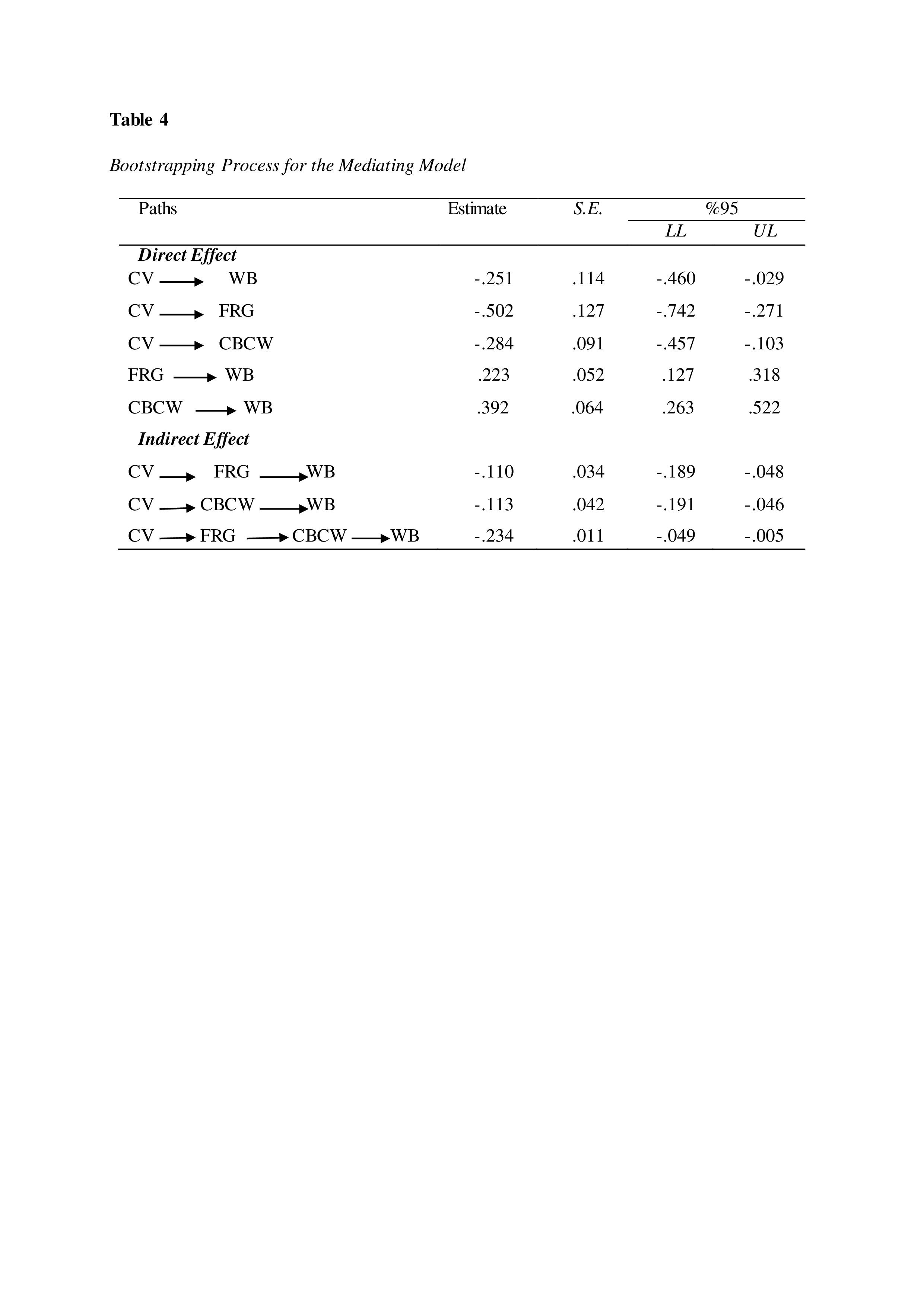

Supplement: Supplementary file 4 [file Image_4.JPEG]
